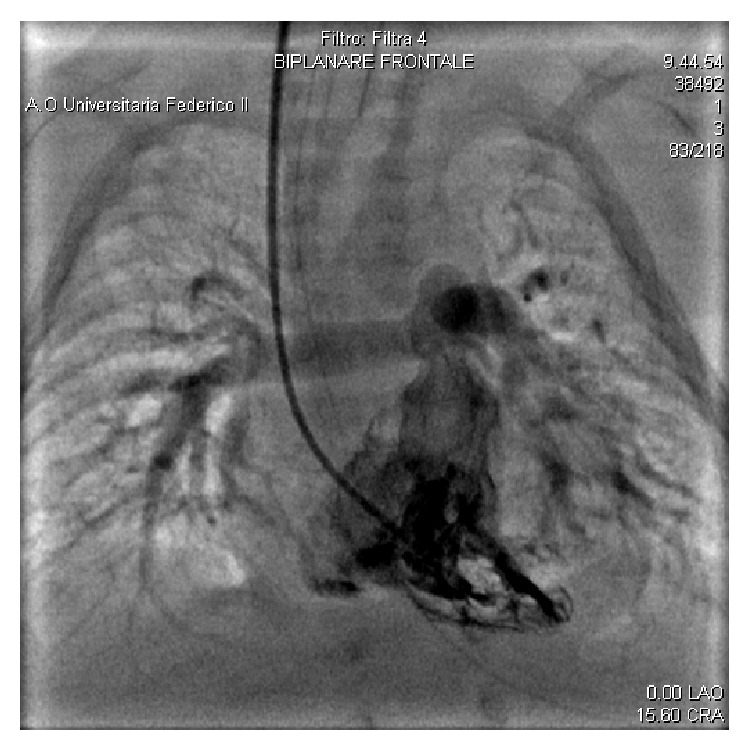# Nitric Oxide-Sensitive Pulmonary Hypertension in Congenital Rubella Syndrome

**DOI:** 10.1155/2015/198570

**Published:** 2015-02-16

**Authors:** Francesco Raimondi, Fiorella Migliaro, Elisa Di Pietro, Francesco Borgia, Antonio Rapacciuolo, Letizia Capasso

**Affiliations:** ^1^Division of Neonatology, Section of Pediatrics, Department of Translational Medical Sciences, Università “Federico II”, Via Pansini 5, 80131 Naples, Italy; ^2^Division of Cardiology, Department of Advanced Biomedical Sciences, Università “Federico II”, Via Pansini 5, 80131 Naples, Italy

## Abstract

Persistent pulmonary hypertension is a very rare presentation of congenital virus infection. We discuss the case of complete congenital rubella syndrome presenting at echocardiography with pulmonary hypertension that worsened after ductus ligation. Cardiac catheterization showed a normal pulmonary valve and vascular tree but a PAP = 40 mmHg. The infant promptly responded to inhaled nitric oxide while on mechanical ventilation and was later shifted to oral sildenafil. It is not clear whether our observation may be due to direct viral damage to the endothelium or to the rubella virus increasing the vascular tone via a metabolic derangement.

## 1. Introduction

Congenital rubella syndrome (CRS) results from an invalidating vertical infection originally described by Gregg as the association of cataracts, deafness, and cardiac defects. CRS is nowadays infrequent in developed countries since the introduction of an effective vaccine preparation.

Persistent pulmonary hypertension of the newborn (PPHN) is a life threatening condition mostly occurring with multiple severe neonatal conditions but hardly ever associated with congenital viral infection. Recently, an interesting Vietnamese series of infants affected from congenital rubella was published, some of whom had developed PPHN [1]. Unfortunately, the pulmonary hypertension onset was not always clear, cardiac catheterization data were unavailable, and no treatment options were given. We discuss the case of an infant affected from full CRS presenting at birth with PPHN showing a progressive course and requiring emergency inhaled nitric oxide (iNO) and later chronic sildenafil treatment. This case allows us to make some pathogenetic speculations on the rare association of PPHN with CRS.

## 2. Case Report

A primiparous woman was admitted for delivery showing a seroconversion for rubella at 9 weeks of gestation. She delivered at 39 weeks of gestation by C-section a baby girl with an Apgar score 7,9 and a birthweight 2320 grams (<5th centile), length 45 cm (<10th centile), head circumference 32 cm (5–10th centile). An echocardiogram on day of life (DOL) 2 was requested for a loud, grade 3 heart murmur and mild distress. It showed a dilated right ventricle with a mild to moderate tricuspid insufficiency and a large patent ductus arteriosus (PDA) and ventricular septal defect (VSD) both shunting left to right and bidirectionally at times. No lung parenchymal process was noted at the standard chest radiogram. In the following days, the baby was diagnosed with bilateral nuclear cataracts and her lab work revealed the presence of antirubella IgM. Viral serologies for CMV, HSV, and EBV were negative. On DOL 12, while the infant was assisted with noninvasive ventilation and FiO_2_ = 0.3, a follow-up echocardiogram showed further dilatation of the right ventricle, with moderate hypertrophy and decreased contractility. The ductal shunting was bidirectional, the ventricular septum appeared flattened, and the estimated systolic pulmonary arterial pressure (PAP) was 38 mmHg. The infant underwent cardiac catheterization that confirmed the echocardiographic data, also detailing a normal pulmonary valve and vascular tree. The mean PAP was 45 mmHg. After the surgical ligation of the large PDA, strict echocardiographic monitoring failed to show a significant hemodynamic improvement. Indeed, the right ventricular dilatation and hypertrophy kept deteriorating with parallel hypoxemia that required mechanical ventilation and an inspired oxygen fraction = 0.9. A second cardiac catheterization procedure assessed a mean PAP = 40 mmHg and confirmed a normal morphology of the pulmonary valve and vascular tree (Figure 1). The infant was administered iNO at 20 ppm with immediate decrease of her oxygen requirement. She was gradually weaned from iNO to oral sildenafil and discharged home at 4 months. At age of 3 months she showed profound bilateral sensorineural loss from 500 to 4000 Hz by auditory brainstem response audiometry. She undergoes strict echocardiographic monitoring. At the current age of 20 months she is still on oral sildenafil as PPHN rebounds at any too rapid weaning attempt.

## 3. Discussion

Persistent pulmonary hypertension has been described as secondary to acquired viral diseases such as HIV but is a very rare presentation of congenital viral infection. Walter-Nicolet et al. recently reported two CMV neonates presenting with progressive PPHN where iNO was not contributory to the outcome [2]. An endothelialitis of the lung is thought to be the cause of CMV mediated pulmonary hypertension as assessed in the postmortem examination of an adult AIDS patient with massive viral lung involvement [3]. Also for rubella virus PPHN is a very rare presentation though the virus has a clear vascular tropism and vessel malformations (mostly isolated stenoses in different body districts) have been described [4]. Recently, Toizumi et al. published the largest series of CRS affected infants with echocardiographic evidence of PPHN [1]. They speculate that volume overload might have generated the pulmonary hypertension since it improved after PDA ligation in some of their patients. The lack of direct imaging in their study opens the possibility that rubella virus might directly interact with the pulmonary vascular tree. Our case points towards this direction since the pulmonary valve and tree had a normal morphology, without significant local stenoses, and the PDA ligation only partially reduced pulmonary hypertension without preventing further deterioration of PPHN. Rubella virus may then exert a cytopathic vascular damage by cellular apoptosis remodeling the terminal arterioles [5]. As an alternative explanation we speculate that (local) infection of rubella virus may interact with cellular metabolism resulting in an increased vascular tone without necessarily killing the host cell. Recently published reviews describe that PPHN may also be due to impaired relaxation of the pulmonary vasculature [6, 7] and it has been demonstrated that the replication of rubella virus in the endothelium can occur without damaging the cell [8]. This theory is also in keeping with the prompt response to iNO and the current dependence on oral sildenafil.

In conclusion, the rare and puzzling presentation of PPHN in CRS may represent a long-lasting threat; its precise mechanism needs to be clarified in individual cases in order to be able to tailor treatment accordingly.

## Figures and Tables

**Figure 1:**